# Supplementary material for: Coupling Up: A Dynamic Investigation of Romantic Partners’ Neurobiological States During Nonverbal Connection
Source: Behav Sci (Basel). 2024 Nov 26;14(12):1133. doi: 10.3390/bs14121133 (PMC11673026; doi:10.3390/bs14121133)

Supplemental Information

1.1 Post-experiment responses to "What were you thinking about during this task?"

My mind was wandering during the eye gaze one. It seems hard to start into someone's eyes for so many minutes. I was thinking how different our relationship may look compared to new ones.
Eyes close holding hands: I was thinking he & I should go see a movie soon I mostly just thinking about my partner & I had a song stuck in my head .
I was thinking about [NAME]. I wondered how long she would keep smiling at me when we had our eyes open. I wanted to make her laugh. I wanted us to communicate somehow. During our embrace, I just kept thinking about how we've perfected our hug. Everything fits.
I was thinking about controlling my breathing with my partner and calming myself.
I kept hoping my eyes wouldn't open. I caught myself sometimes.
[NAME] heart beat, his chest falling up & down, smell of his shirt.
I was thinking about how much I love my partner and how happy I am to be here doing this study with him. I was also thinking about things that make me laugh because I'm bad at holding still.
How goofy we both are. A ton of things were going on in my head. Giving daily thanks and a specific song. When our eyes opened I thought about how it was impossible to keep a straight face.
I was thinking about loving my partner/wanting to kiss him :)
I was thinking what are we doing. I wasn't thinking anything specific. I was wondering when it might end.
Hugging - I was thinking about what I will eat for dinner. Eye contact – my partner and I having different comfort levels with eye contact and that he looked tense. I also laughed when he laughed. Hand-holding - I was noticing how much my partners moves his hands.
[NAME] laughs too much. Combination of remembering every reason why I love him & why I'm in this study. Also distracted by my mind getting distracted w/the changing image of his face when I stared at it for too long. I also realized I think a lot! Closing my eyes helped me feel more focused & connected.
I was just wondering what my partner was thinking about honestly.
When I had to stare in to my partners eyes I had trouble not laughing because of the EEG head cap and I could tell [name] wanted to laugh too. I was focused on the holding hands and hugging tasks. Thought about [name] and our connection.
I was thinking about our relationship and some memories of us together. Also thought about us dancing together.
Some – how he looked with the cap on, -the funny faces he was making. Later on – how warm his hand felt, how long we've been together
I was thinking about not falling over because [NAME] kept leaning back. Also I wanted to rest my head on his shoulder, but I was worried about the EEG.
Simply how happy I am at this point in my life. I don't need anything else (hugging + holding hands). When my eyes were closed, my mind went to unrelated things.

Often times thinking about my partner. Also thinking about school work and what I'm going to do when I get home.
The 1st one: closed eyes, no touch – I had a big urge to reach out & touch [name]. And then when were hugging, I was envisioning how it'll feel on our wedding day (we talk of getting married in [PLACE] w/close friends & family) & I was picturing it <3
What is this measuring?
I was thinking about how we hugged for a long time like this on our wedding day
Why doesn't [NAME] smile looking @ me ? He makes me giggle, will that mess anything up? What part(s) of my face is he looking at? Why are my hands on top? If we switched during the eye open test, would the results look different? He smells like natural [NAME] smell. We are both wearing navy tops, might look like a navy blob
Trying to focus but have a difficult time. Trying to hear my partner during the first task, and occasionally making a breath sound to be heard by my partner. Occasionally thinking about needing to use the restroom... Wondering what my partner thought I was thinking. Thinking the physical contact was pleasant. Thinking about not laughing while my partner was laughing
I was trying to think about [NAME] so I was remembering a lot of the great moments we have had together in the past 17 years of our relationship.
When my eyes were closed I would think about what I was hearing. The activities where I was looking at [NAME] I thought about how his facial expressions would make me laugh more.; The hugging one I thought about how cuddle-y [NAME] hugs were and how he smelt good.
Counting down mostly, and a few random thoughts.
I was thinking about how [NAME] was not holding me tightly. I was wondering why she didn't.
I was thinking about how sexy and beautiful she looks and how special she is to me. I love that woman.
For the first bit I sang "Coward of the County" by Kenny Rogers in my head. Then I tried to make a game out of getting reactions from [NAME] by making faces at her.

1.2 Post-experiment responses to "What were you feeling during this task?"

Pressure to make sure I was being loving enough.
Very calm
I felt peaceful. At ease
I was feeling relaxed. I wanted to close my eyes and feel the hug and embrace.
I felt calm. Sometimes I felt like our breathing was becoming in-sync when we were embracing, touching, and looking at each other.
Safe & comfortable; Pleasant
I felt happy and excited. I felt loved by my partner.
I felt a little silly and immature. The hug felt nice and relaxing compared to the blank stare.
Feeling love/happiness
It felt weird. I felt stupid
I felt relaxed.
Combination of love & comfort – almost unconscious comfort – like the ability to breathe or think. also, I felt like I was trying hard to focus on our assigned task.
I felt joy and connection between the two of us for sure.
I was feeling awkward at first on the staring task but slowly I felt more admiration and compassion for my partner thereafter.
Love for my partner. Safe and content
Initially, kind of embarrassed, funny. Later, warm & comfortably fuzzy
Safe and warm and protected.
Love. My love for my partner and her for me.
Relaxed.
Love! So much love for him & when we looked at each other I couldn't keep from smiling. My cheeks hurt!
Happy to hug [NAME]; Curious what yawning looked like on the brain scanner
I felt relaxed
Insecure, Curious, Anxious, Silly
Connected to my partner by the shared experience. Comfortable during the embrace. Nervous about focusing
I was often feeling awkward because I know we are being observed, but I was also feeling very positive about my relationship with [NAME]
When staring at [Name] I felt awkward because he kept chuckling, but the hug one I felt very comfortable and relaxed. When I closed my eyes I felt at ease & peaceful
A little happy, a little impatient and very weird.
I felt lucky and fortunate to have her in my life
Feeling loved
Bored, frustrated, want to be done

Table S1. Daily items from evening survey: Note: All scales are recoded such that "1 = strongly disagree" and 10 = "strongly agree".

Construct	Question	Reverse coded
Perception of love	How much do you feel loved by your partner today?	no
Loving feelings	My partner and I were close and connected with each other today.	no
Loving feelings	My partner and I were loving and affectionate with each other today.	no
Negative feelings	My partner and I were angry or mad at each other today.	yes
Negative feelings	There was tension between my partner and me today.	yes
Positive feelings	My partner and I showed appreciation and/or gratitude for each other in some way today.	no
Positive feelings	My partner and I complimented or said something positive about the other to each other today.	no
Wellbeing	You felt healthy today.	no
Wellbeing	You had enough energy today.	no
Wellbeing	You did well at work today.	no
Wellbeing	Your co-workers were pleased with you today.	no
Wellbeing	You had a good time playing with friends today.	no
Wellbeing	You had a good time with family members today.	no
Wellbeing	You played with your pet today.	no
Wellbeing	You enjoyed the time for entertainment, recreation, hobbies, or activities today.	no
Wellbeing	You learned something new today.	no
Wellbeing	You felt close and connected with other people today.	no
Wellbeing	You were lonely today.	no
Wellbeing	You did what interests you today.	no
Wellbeing	You had to do things against your will today.	no
Wellbeing	You successfully completed difficult tasks today.	no
Wellbeing	You experienced some kind of failure today.	no
Wellbeing	You were "real" and authentic today.	no
Wellbeing	You were your true self today.	no
Quantity of shared time	How much time did you spend together?	
Quantity of shared time	How much time did you communicate with your partner?	

Table S2. Descriptive statistics of concordance correlation coefficients (CCC) for each condition at the subject-level. CCC values were computed for each subject in reference to their partner and are described as collapsed across persons.

Condition	Lag	Mean	SD	Min	Max	Skew	Kurtosis
**No Connection**	Concurrent	-0.005	0.080	-0.191	0.090	-0.981	0.105
**Gaze Only**	Concurrent	-0.027	0.096	-0.192	0.146	-0.339	-0.742
**Hands only**	Concurrent	0.028	0.069	-0.073	0.192	0.752	-0.074
**Gaze and Hands**	Concurrent	-0.004	0.064	-0.168	0.129	-0.438	1.265
**Embrace**	Concurrent	-0.029	0.087	-0.248	0.091	-0.999	0.538
**No Connection**	Lag 1	-0.009	0.072	-0.246	0.111	-1.247	2.321
**Gaze Only**	Lag 1	-0.024	0.076	-0.185	0.125	0.110	-0.449
**Hands only**	Lag 1	0.012	0.065	-0.097	0.177	0.658	0.241
**Gaze and Hands**	Lag 1	-0.011	0.077	-0.238	0.106	-0.948	0.711
**Embrace**	Lag 1	-0.004	0.106	-0.239	0.293	0.961	2.120
**No Connection**	Lag 2	0.058	0.136	-0.161	0.389	0.660	0.104
**Gaze Only**	Lag 2	-0.022	0.167	-0.682	0.228	-2.073	5.866
**Hands only**	Lag 2	0.022	0.130	-0.213	0.330	0.115	-0.247
**Gaze and Hands**	Lag 2	0.050	0.104	-0.088	0.404	1.409	2.400
**Embrace**	Lag 2	-0.026	0.138	-0.417	0.261	-0.480	0.719
**No Connection**	Lag 3	-0.005	0.095	-0.254	0.318	0.792	3.477
**Gaze Only**	Lag 3	-0.023	0.078	-0.246	0.066	-1.201	0.599
**Hands only**	Lag 3	-0.017	0.070	-0.179	0.140	-0.485	0.235
**Gaze and Hands**	Lag 3	0.022	0.063	-0.105	0.199	0.480	0.417
**Embrace**	Lag 3	-0.004	0.064	-0.128	0.138	0.172	-0.236

Figure S1. Relationship between FAA and baseline characteristics. FAA values are extracted as each person's average FAA value during Gaze and Hand condition, as the condition by which FAA was positive, reflecting more approach-like neurobiological states. Individual difference factors are computed as the mean from baseline surveys at the study onset. For panels 5-6, Specific questions are available in Table S1, and items are scored from strongly disagree (1) to strongly agree (10).

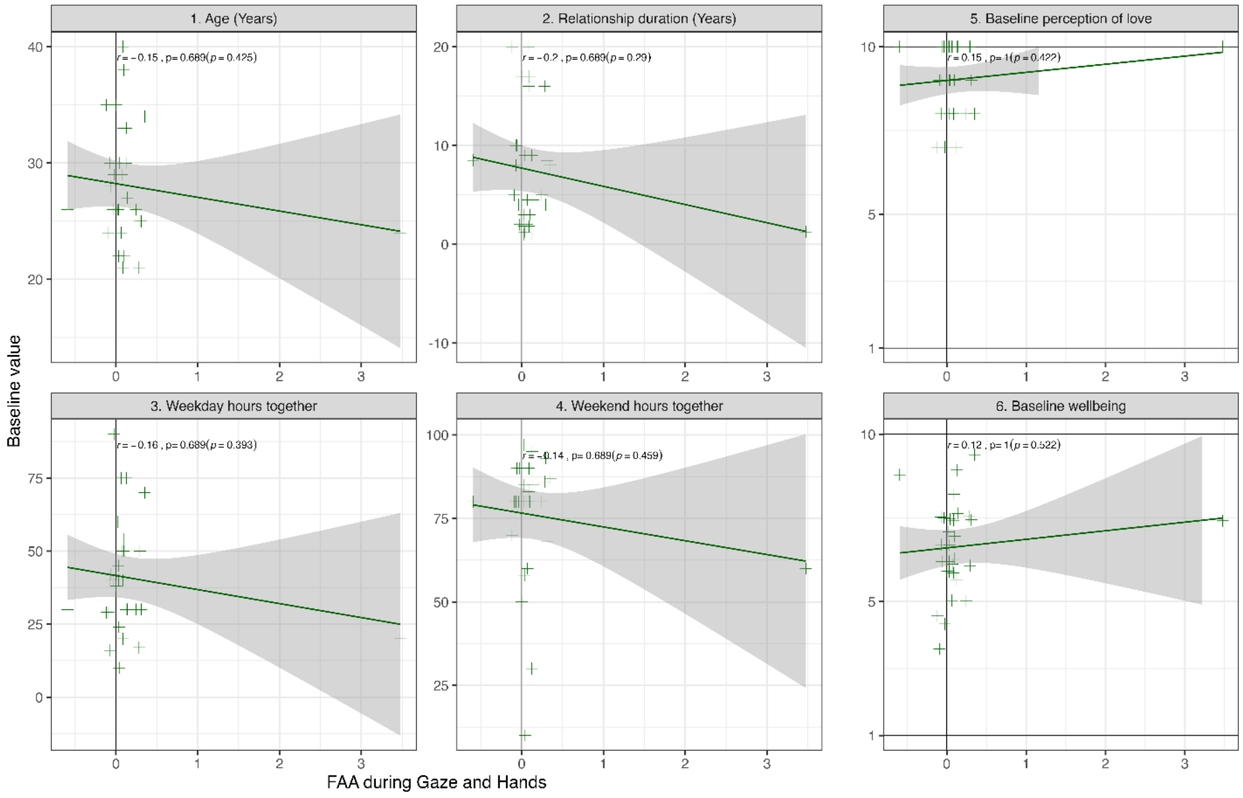

Figure S2. Relationship between concordance and baseline characteristics. CCC values are extracted as each person's average CCC value during Gaze and Hand condition during Lag 2. In this way, positive values indicate concordance between the person's FAA value and their partner's FAA value two bins previous (i.e., 4 seconds ago). Individual difference factors are computed as the mean from baseline surveys at the study onset. For panels 5-6, Specific questions are available in Table S1, and items are scored from strongly disagree (1) to strongly agree (10).

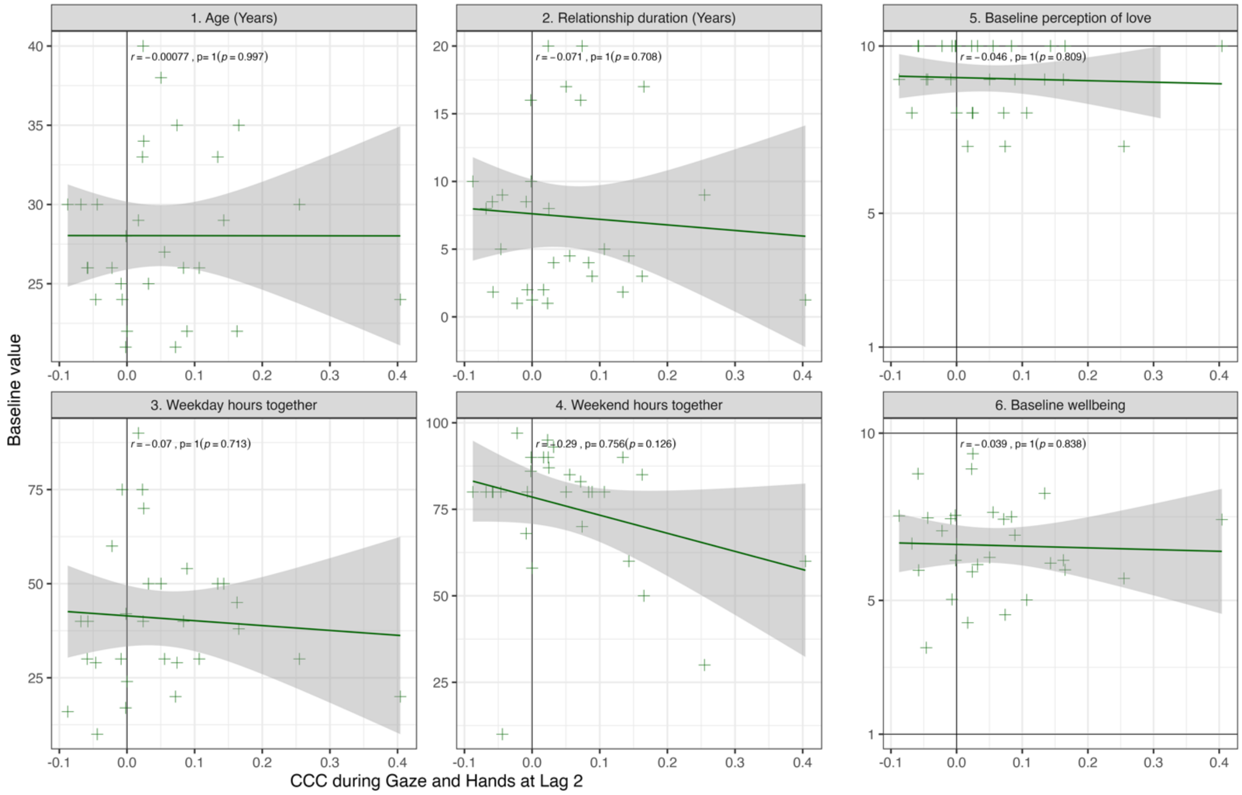

Figure S3. Relationship between concordance and average weekly experiences. CCC values are extracted as each person's average CCC value during Gaze and Hand condition during Lag 2. In this way, positive values indicate concordance between the person's FAA value and their partner's FAA value two bins previous (i.e., 4 seconds ago). Individual difference factors are computed as the weekly means from daily evening surveys and specific questions are available in Table S1. For panels 1-5, items are scored from strongly disagree (1) to strongly agree (10).

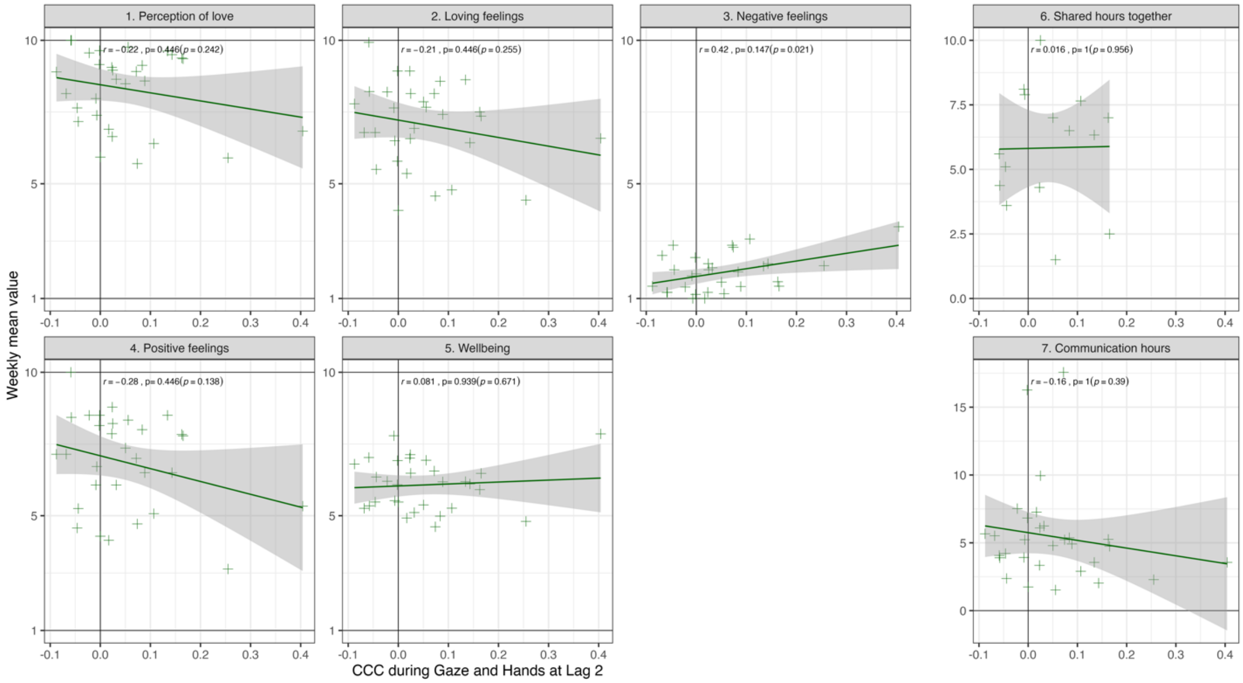

Figure S4. Relationship between FAA and baseline characteristics with winsorized outliers. FAA values are extracted as each person's average FAA value during Gaze and Hand condition, as the condition by which FAA was positive, reflecting more approach-like neurobiological states. Individual difference factors are computed as the mean from baseline surveys at the study onset. For panels 5-6, Specific questions are available in Table S1, and items are scored from strongly disagree (1) to strongly agree (10).

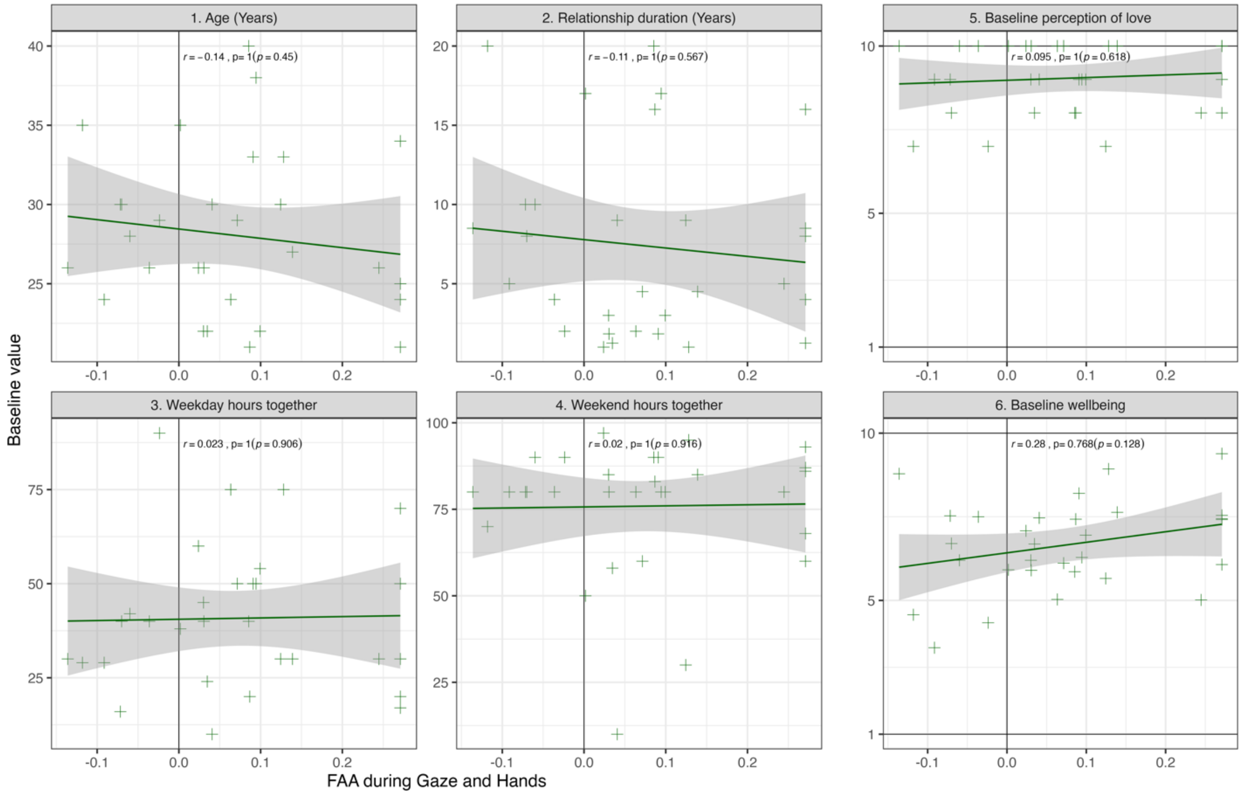

Figure S5. Relationship between FAA and average weekly experiences with winsorized outliers. FAA values are extracted as each person's average FAA value during Gaze and Hand condition, as the condition by which FAA was positive, reflecting more approach-like neurobiological states. Individual difference factors are computed as the mean from baseline surveys at the study onset. For panels 5-6, Specific questions are available in Table S1, and items are scored from strongly disagree (1) to strongly agree (10).

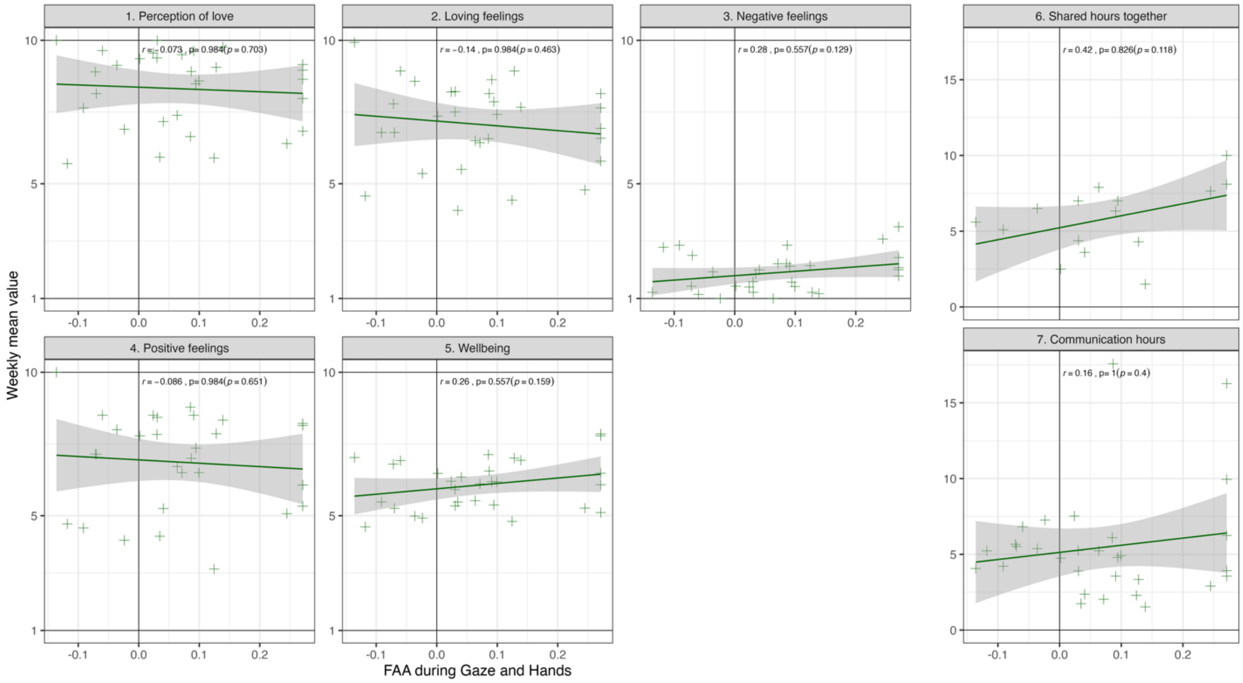

Figure S6. Relationship between concordance and baseline characteristics with winsorized outliers. CCC values are extracted as each person's average CCC value during Gaze and Hand condition during Lag 2. In this way, positive values indicate concordance between the person's FAA value and their partner's FAA value two bins previous (i.e., 4 seconds ago). Individual difference factors are computed as the mean from baseline surveys at the study onset. For panels 5-6, Specific questions are available in Table S1, and items are scored from strongly disagree (1) to strongly agree (10).

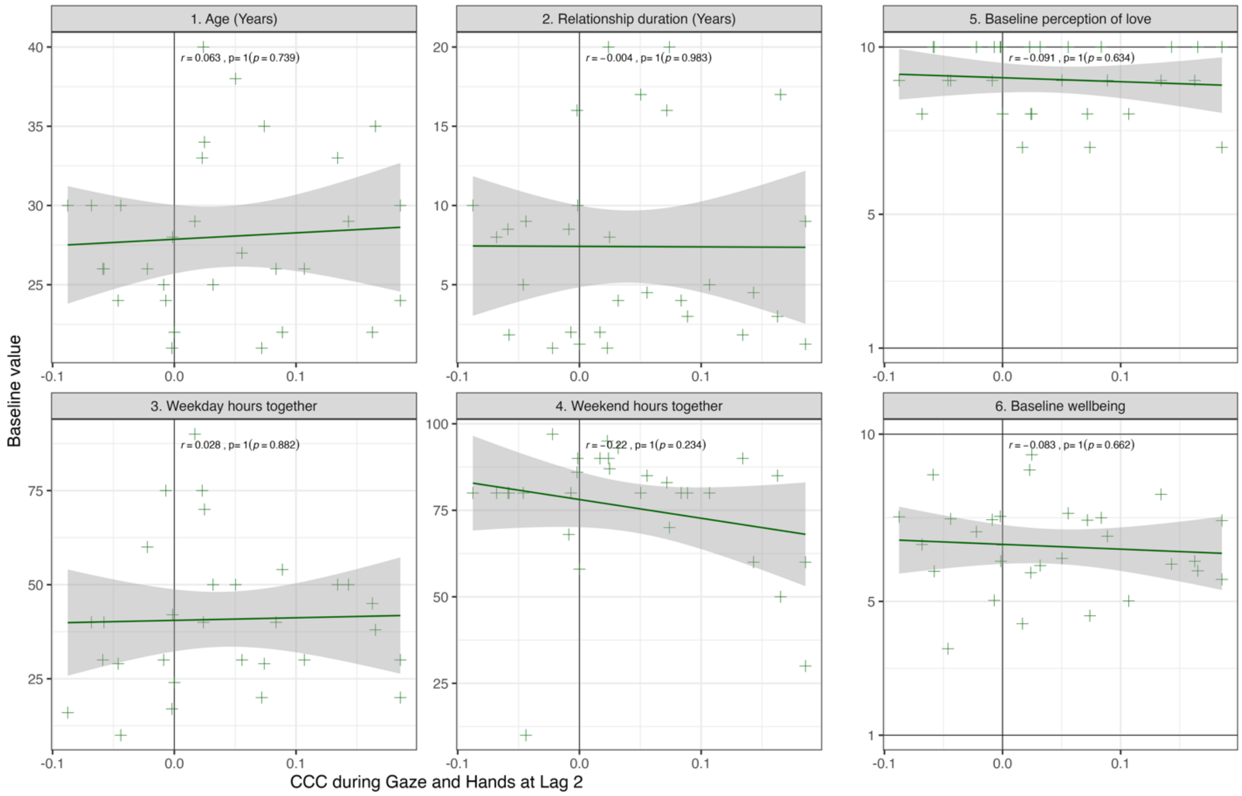

Figure S7. Relationship between concordance and average weekly experiences with winsorized outliers. CCC values are extracted as each person's average CCC value during Gaze and Hand condition during Lag 2. In this way, positive values indicate concordance between the person's FAA value and their partner's FAA value two bins previous (i.e., 4 seconds ago). Individual difference factors are computed as the weekly means from daily evening surveys and specific questions are available in Table S1. For panels 1-5, items are scored from strongly disagree (1) to strongly agree (10).

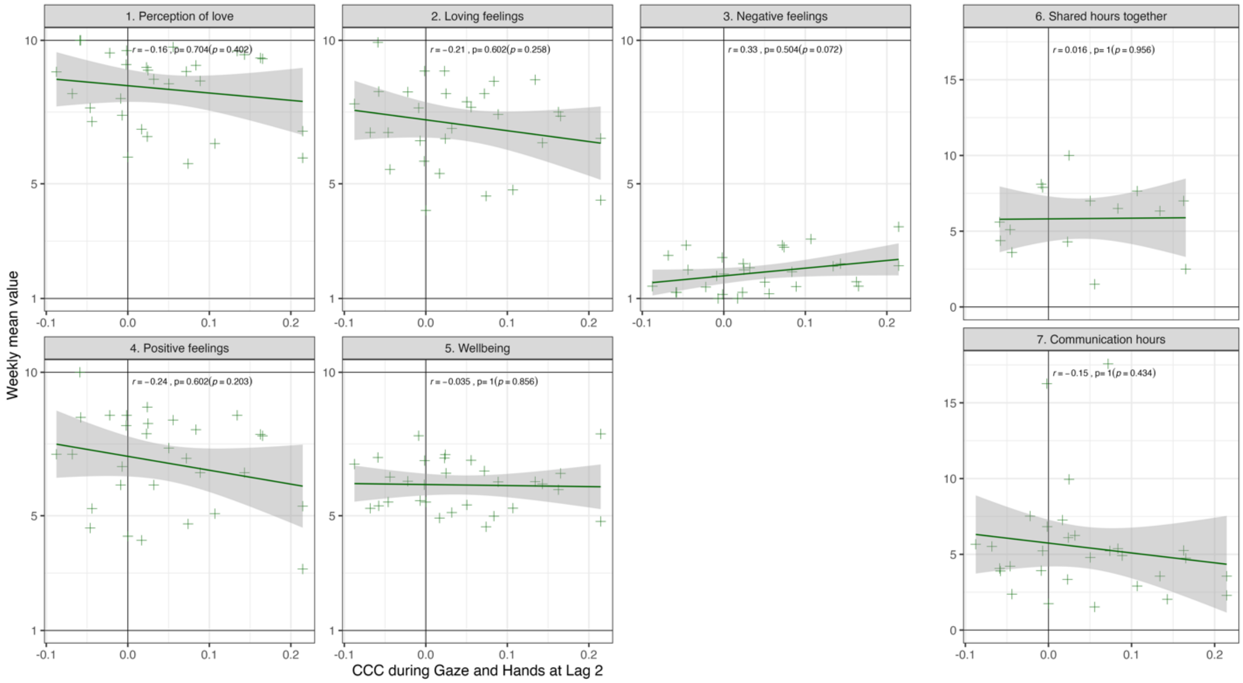

Supplement: Supplementary file 1 [file behavsci-14-01133-s001.zip › behavsci-3255517-supplementary.pdf]
